# Efficacy and safety of inhaler steroids in COPD patients: Systematic review and meta-analysis of randomized placebo-controlled trials

**Published:** 2014

**Authors:** Reza Karbasi-Afshar, Jafar Aslani, Mostafa Ghanei

**Affiliations:** 1 Atherosclerosis Research Center, Baqiyatallah University of Medical Sciences; Tehran; 2 Chemical Injuries Research Center, Baqiyatallah University of Medical Sciences; Tehran

**Keywords:** Inhaler steroid, COPD, Systematic review

## Abstract

***Background: ***Chronic obstructive pulmonary disease (COPD) is a major health dilemma and cause of morbidity and mortality in either industrialized or developing countries and inhaled corticosteroids are widely used worldwide in these patients. In this systematic review, we aimed to analyze the effectiveness of these gents compared to placebo.

***Methods:*** Pubmed and Google Scholar literature search has been done to find randomized placebo-controlled trials investigating effectiveness of inhaled steroids in COPD patients. Finally, the data from 18 trials had been retrieved and included into a database, and analyzed using Stata ® v.9.0.

***Results:*** Data of 12, 297 COPD subjects were analyzed. Analysis of survival of patients in the two groups showed no significant difference between those taking inhaled corticosteroids or placebo (relative risk (RR): 1.071, 95% confidence interval (CI): 0.938-1.224, P=0.309). Patients taking inhaled steroids were significantly less likely to develop an exacerbation episode (RR: 0.697, 95%CI: 0.596-0.816, p<0.001) or to have less withdrawal rate than placebo (RR: 0.882, 95%CI: 0.811-0.960; P=0.004).

***Conclusion: ***Because steroid inhalers represent no survival effects in COPD patients, and due to their life threatening side effects (pneumonia, candidemia, etc.), we propose to replace steroid inhalers to cheaper agents which provide patients with comparable advantages (e.g. few exacerbations) and fewer side effects. Pulmonary rehabilitations as well as anti-inflammatory drugs are recommended to be more attended in COPD patients.

Chronic obstructive pulmonary disease (COPD) is a major health dilemma and cause of morbidity and mortality in either industrialized or developing countries. This disease is commonly associated with therapeutic side effects and exacerbations, which significantly affect patients’ health and quality of life (1), and they can even significantly increase the mortality (2). For managing COPD effects, various types of therapeutic agents have been employed from which bronchodilators including β2-agonists and anticholinergics are usually considered inevitably included in the standard treatment for COPD exacerbations (3). Inhaled corticosteroids used alone or in combination with long-acting β2-agonists have been widely used in the treatment of patients with asthma, and evidence is highly suggestive of their beneficial effects in asthmatic patients. Nonetheless, despite the difference in the pathogenesis, the same therapeutics have been widely used for COPD patients, particularly in patients with moderate to severe disease (4). 

Despite the presumptions on the outcome benefits of inhaled corticosteroids in the management of COPD, recent evidence coming from randomized trials has put high levels of controversy on the efficacy of these agents (5). In order to evaluate the feasibility of these agents on reducing unfavorable effects of COPD, in a recent meta-analysis we showed that long-acting β agonists, despite their favorable effects on exacerbation rates and tolerability of the drug, they represent no survival advantage over placebo [unpublished data]. We explained this observation with either absence of any outcome advantage for these drugs in COPD treatment or its serious side effects, particularly cardiovascular, that adversely affect their outcome. In the current study, we systematically reviewed the existing literature to find randomized placebo controlled trials investigating the effects of inhaled corticosteroids comparing them to those of inhaled placebo and analyze their pooled data to find any potential significant effects for inhaled steroids in COPD patients. 

## Methods

To conduct our systematic review, the primary search was done using the terms *"inhaled corticosteroids”,* “*COPD*” and “*randomized controlled trial*” as the keywords in the time span 1995-2013. A repeat of the search using keywords, *“budesonide”* or “*fluticasone*” instead of *"inhaled corticosteroids”*, was performed to expand the included studies. The literature search was repeated again using the terms “*inhaled corticosteroids*” and “*efficacy*” or “*safety*” or “*exacerbation*” or “*withdrawal*”/* “discontutation”* and “*COPD*” “*randomized controlled trial*”. Pubmed database was used as the initial search instrument, which we believe it relatively provides the largest published data of the most relevant studies in the fields of respirology. Then the search has been strengthened searching the citations of the found articles in the Google Scholar to find potential publications which have neither been indexed in Pubmed nor retrieved through Pubmed search. In our search, overall 94 studies were found upon a search of the literature via Pubmed search using the mentioned keywords. Then, the found titles and the abstracts of the studies were screened to find the appropriate studies associated with our systematic review, and randomized controlled trials. Finally, 18 randomized controlled trials investigating the efficacy and safety of budesonide (7 trials), fluticasone (10 trials) or mometasone furoate (1 trial) on the disease course, drug tolerance and survival of COPD patients were enrolled into a meta-analysis (table 1) (4, 6-22). The analyses were performed on three major study variables: exacerbations, drug withdrawal and patient’s survival. The meta-analysis has been performed using software Stata v.9.0 (Stata corp, TX, USA). 

**Table 1:** Randomized placebo controlled trials included in the current meta-analysis

**No.**	**Name of Author**	**Reference**	**Year**	**Placebo group (n)**	**Steroid group (n)**	**Inhaled steroid type**
1	DIMCA study	[6]	2003	24	24	Fluticasone
2	Vestbo et al.	[7]	1999	118	128	Budesonide
3	Szafranski et al.	[8]	2003	205	198	Budesonide
4	Renkema et al.	[9]	1995	18	21	Budesonide
5	Paggiaro et al.	[10]	1998	139	142	Fluticasone
6	Calverley et al.	[11]	2003	361	374	Fluticasone
7	Burge et al.	[12]	2000	375	376	Fluticasone
8	Bourbeau et al.	[13]	1998	40	39	Budesonide
9	Albers et al.	[14]	2004	25	24	Fluticasone
10	Calverley et al.	[15]	2007	1524	1534	Fluticasone
11	Hanania et al.	[16]	2003	185	183	Fluticasone
12	Calverley et al.	[17]	2003	256	257	Fluticasone
13	Jenkins et al.	[18]	2009	1524	1534	Fluticasone
14	Pauwels et al.	[19]	1999	643	634	Budesonide
15	Mahler et al.	[20]	2002	181	168	Fluticasone
16	Tashkin et al.	[21]	2012	448	463	Mometasone furate
17	Doherty et al.	[4]	2012	236	253	Mometasone furate
18	Maltais et al.	[22]	2002	56	63	Budesonide

## Results

Data of 12,297 COPD subjects including 6173 patients under an inhaled corticosteroid and 6124 patients taking inhaled placebo retrieved from 18 randomized controlled trials and were enrolled into this meta-analysis. 4616 COPD patients were under fluticasone, 1304 were taking budesonide and the remaining 253 patients were under mometasone furate therapy. 

**Figure 1:**
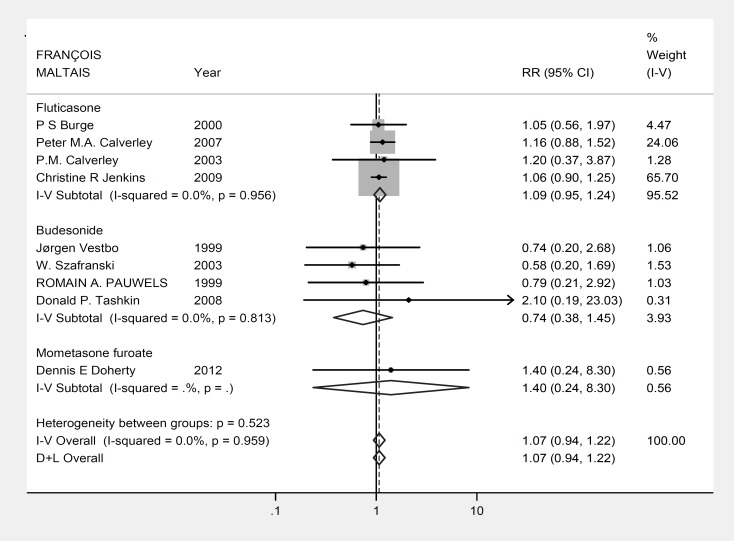
Forest plot: Meta analysis effects of inhaled steroids on COPD patients survival

**Analysis of survival:** Figure 1 summarizes the analysis of data. Analysis of survival of patients in the two groups showed no significant difference between those taking inhaled and placebo (relative risk (RR): 1.071, 95% confidence interval (CI): 0.938-1.224, P=0.309, z= 1.02; figure 1). No significant heterogeneity has been observed among the survival data of the included studies, indicating a high reliability value for the analysis (P=0.959; heterogeneity χ^2^=2.57 (df=8) I^2^= 0.0%). 

**Figure 2:**
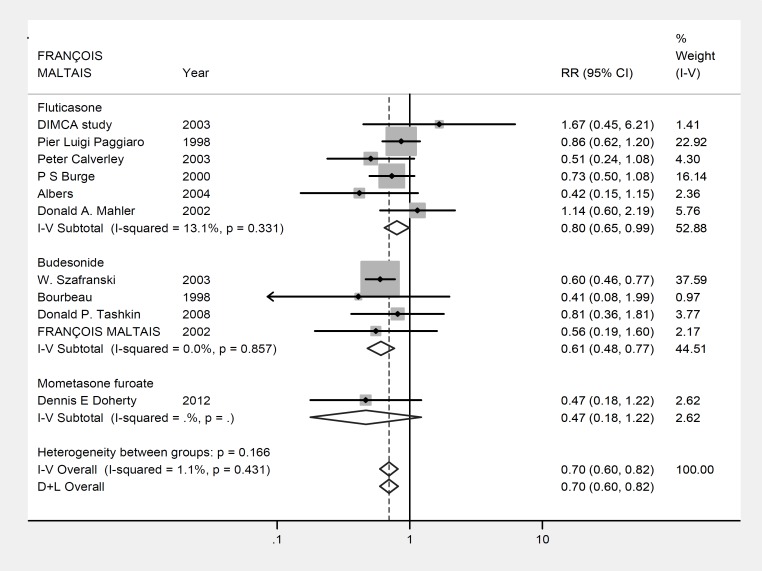
Forest plot: Meta-analysis effects of inhaled steroids on COPD disease exacerbations

**Exacerbations:** Figure 2 summarizes the analysis of data. Analysis of the rates of the patients experiencing exacerbation episodes within the trial period, however, showed that COPD patients taking inhaled steroids were significantly less likely to develop an exacerbation episode (RR: 0.697, 95%CI: 0.596-0.816, p<0.001, z=4.5; figure 2). The heterogeneity of the included studies in the exacerbation rate was significantly high (p=0.007, Heterogeneity χ^2^ = 10.11 (df=10) I^2^= 1.1%). 

**Figure 3:**
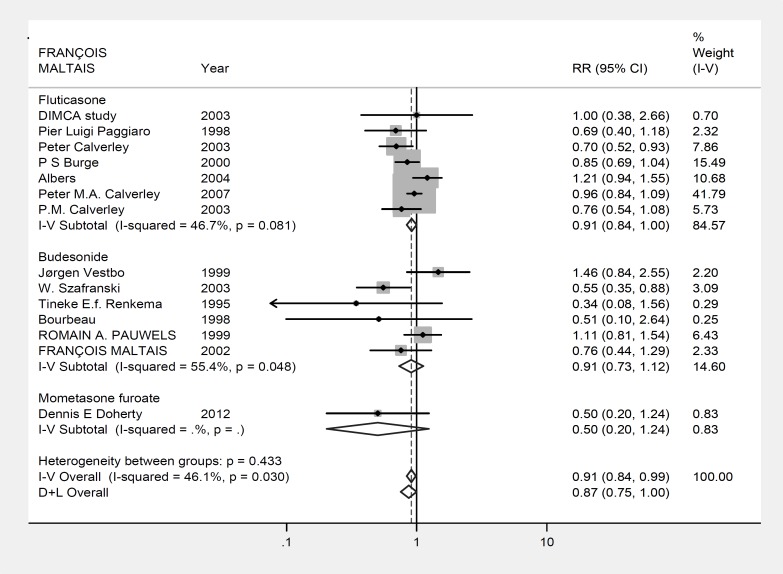
Forest plot: Meta-analysis effects of inhaled steroids on tolerance to therapy by COPD patients

**Tolerance to therapy:** Figure 3 summarizes the analysis of data. Similar observation was detected in analyzing the rate of drug withdrawal in patients of the two groups with patients under placebo having significantly a higher rate of drug discontinuation due to adverse events or disease symptoms (RR:0.882, 95% CI: 0.811-0.960; P=0.004, z= 2.89; figure 3). 

However, unlike what we observed in the analysis of survival and/or exacerbations, the heterogeneity rate was significantly high for tolerance to the therapy (P=0.026, heterogeneity χ^2^ = 24.59 (df=13) I^2^= 47.1%).

## Discussion

This review and meta-analysis of 18 randomized placebo-controlled trials of ICS therapy in COPD patients revealed several important findings. This study demonstrated that inhaled corticosteroids used for the management of COPD are not able to improve survival of the patients, while they can significantly improve exacerbation rate and they also would be significantly better tolerated by COPD patients. The findings of the current study raise important questions over the current therapeutic strategy for COPD patients which presumably include inhaled steroids in their regimens. 

The finding of this study that inhaled steroids have no significant survival effect in COPD patients is consistent with some previous meta-analyses (23-25). The current systematic review and meta-analysis using updated data including more recent data from the literature corroborated the findings from the previous meta-analyses. In a previous meta-analysis [unpublished data] of randomized placebo controlled trials investigating potential effects of β_2_-agonists on COPD patients’ outcome, we also reached to similar findings for this agent groups, with no survival effect on them. Putting together, since traditional treatment protocols for COPD include inhaler beta agonists and steroids, we believe that these data suggest that the therapeutic strategies employed to manage COPD do not promise outcome advantages and new approaches are needed to control the disease in this patient population. 

As mentioned before, this meta-analysis, however, showed some beneficial effect for inhaled steroids in reducing the rate of exacerbation episodes in COPD patients. This finding is of high importance, because we know that exacerbations do not only affect quality of life of COPD patients, but also can increase mortality (26, 27). Nevertheless, some recent studies have proposed similar effects for exercising and pulmonary rehabilitation in COPD patients. Seymour et al. (28) in their trial of 3 months found that exercise can reduce execrbation rates in COPD patients. Similar favorable effects have also been detected on the quality of life of COPD patients undergoing pulmonary relhabilitation (29). 

There is even suggestive line of evidence of survival benefits of rehabilitation for COPD patients (30). So, the authors of the current article believe that inhaled steroids due to non-significant survival effects as well as the significant rate of side effects are not suitable choices for COPD management, and can be safely substituted by less costly and safer therapeutic strategies like exercise. Moreover, this study showed a better tolerance to inhaled steroids representing lower withdrawal rate than placebo. This observation is also suggestive of some relieving effect for this drug on COPD disease burden, and may be suggestive of some quality of life advantage. However, as mentioned before, this range of advantages would not cover the disadvantages of the drug’s side effects, and when we could use rehabilitation therapy with comparable benefits and fewer deleterious effects; there would not be any indication for administration of them. Moreover, there is increasing evidence for the beneficial effects of anti-inflammatory drugs like theophylline in COPD patients which may be able to promise better outcome with fewer side effects than inhaled steroids (31). This study has limitations. Most of the studies were not following their patients in a long-term period, and mortality was investigated as an incident event and not as the final end point, and due to the limited follow up time in most of the studies as well as population sizes, they might not be a good representative of associated mortality in these populations. This may put a serious restriction on the capacity of the current analysis to judge on the potential beneficial or deleterious effects of inhaled steroid therapy in COPD patients. 

In conclusion, because steroid inhalers represent no survival effects in COPD patients, and also due to their confirmed life threatening side effects (pneumonia, candidemia, etc.), we propose to replace steroid inhalers with cheaper agents which provide patients with comparable advantages (e.g. few exacerbations) and fewer side effects. Pulmonary rehabilitations as well as anti-inflammatory drugs are recommended to be more attended in COPD patients. 

## Notes

**Funding:** This study has been funded by Baqiyatallah University of Medical sciences; and no financial support from pharmaceutical companies.
